# Core Temperature Response During the Marathon Portion of the Ironman World Championship (Kona-Hawaii)

**DOI:** 10.3389/fphys.2019.01469

**Published:** 2019-12-03

**Authors:** Guillermo Olcina, Carmen Crespo, Rafael Timón, Jeffrey M. Mjaanes, Julio Calleja-González

**Affiliations:** ^1^Research Group in Sport Training and Physical Conditioning (GAEDAF), Faculty of Sport Sciences, University of Extremadura, Cáceres, Spain; ^2^Department of Orthopaedics, Northwestern University, Chicago, IL, United States; ^3^Department of Physical Education and Sports, University of the Basque Country (UPV-EHU), Vitoria, Spain

**Keywords:** endurance, performance, competition, hyperthermia, triathlon, marathon

## Abstract

The Ironman triathlon consists of a 3.8 km swim, 180 km bike, and 42.195 km run. Thermoregulation responses play an important role in performance optimization and injury prevention. Factors such as environmental conditions including heat and humidity, athlete training level, and race duration can affect thermoregulation. Hyperthermia occurs when the core temperature rises above 38.5°C. The present study aims to describe core temperature (Tcore) in top-level and well-trained age group triathletes during the marathon of Ironman World Championship 2014 in Kona-Hawaii under thermal stress conditions. Tcore of 15 triathletes (age: 36.11 ± 7.36 years, body mass: 71.14 ± 7.12 kg, height: 179 ± 0.04 cm, and fat %: 8.48 ± 0.85) who classified for the Ironman World Championship was measured by an ingestible pill telemetry system prior to competition, during the marathon and 60 min after finishing the race. Mean wet bulb globe temperature (WBGT) during the marathon was 24.66°C (range 22.44–28.50°C). Body mass index (BMI) and perceived exertion (Borg Scale and Visual Analog Scale-Pain) were collected before the race and 60 min after the event. Time variables were extracted from their official race time and split times. Finish time was 10: 06:56 ± 0:48:30. Tcore was initially 36.62 ± 0.17°C, increased at the end of the event (38.55 ± 0.64; *p* < 0.01) and remained elevated 60 min after the event (38.65 ± 0.41°C; *p* < 0.002). BMI significantly decreased after the event (22.85 ± 1.11 vs. 21.73 ± 1.36; *p* < 0.05), whereas both exercise perceived exertion [Borg Scale (10.2 ± 1.64 vs. 18.60 ± 1.67; *p* < 0.003)] and perceived muscle pain [VAS Pain (2.75 ± 1.59 vs. 9.08 ± 1.13; *p* < 0.001)] increased significantly after the event. Tcore during competition correlated negatively with position in age group (*r* − 0.949, *p* = 0.051), but not with race time (*r* = −0.817; *p* = 0.183). High-level age group triathletes competing under thermal stress conditions in the Kona Ironman reached a state of hyperthermia during the marathon. After 60 min of recovery the hyperthermia persisted. Strategies to aid post-event cooling and recovery should be considered to avoid the potentially dangerous adverse health effects of hyperthermia.

## Introduction

A triathlon race involves consecutive sequences of a 3.8 km swim, a 180 km cycle ride, and a 42.2 km run. The Ironman triathlon has gained significant popularity in recent years. During the Ironman triathlon, participants compete for best overall race time, including timed transitions between the swimming, biking, and running legs. The multiple, worldwide Ironman competitions (Lepers, 2008) have become popular as they are accessible to multitudes of amateur and recreational athletes. With the increasing popularity of these competitions, numerous studies have been published in recent years aiming to analyze the physiologic responses of endurance triathletes in competition.

Laursen and colleagues demonstrated that during an Ironman triathlon recreational triathletes’ Tcore reached 38.1 ± 0.3°C (Laursen et al., 2006). Increases in Tcore above 38°C, induced by intense exercise, produce central fatigue (Nybo and Nielsen, 2001); moreover, time-to-exhaustion is reduced in the heat (Gonzalez-Alonso et al., 1999). Additionally, Nybo and colleagues demonstrated that elevated core temperature alters prefrontal cerebral activity (Nybo and Nielsen, 2001). Hyperthermia (H) is defined as an elevation of core body temperature (Tcore) greater than 38.5°C. Exertional heat illness is a spectrum of conditions involving elevated body temperature; the two most serious of these conditions are heat exhaustion and exertional heat stroke (EHS). Core temperature is a critical vital sign in the assessment of a collapsed marathon runner who may have EHS (Ronneberg et al., 2008).

During exercise, Tcore is proportional to the metabolic rate and is largely independent of a wide range of environmental conditions. Accelerated hyperthermia-mediated fatigue during maximal and prolonged exercise is preceded by functional alterations in central nervous and cardiovascular systems as well as in skeletal muscle. Parry and colleagues proposed that impaired marathon running performance in warm environments is associated with greater thermal, cardiovascular, and metabolic strain as well as greater perceived exertion; research demonstrated positive correlations between distance covered and rating of perceived exertion (Parry et al., 2011). These effects prevent marathon runners from competing at their personal record speed without inducing accelerated regulatory dysfunction in multiple bodily systems (González-Alonso, 2007).

A reliable core body temperature is important for differentiating between heat exhaustion and EHS, as well as monitoring the success of cooling therapies. An accurate measurement of Tcore is essential to protect individuals from heat injury during exposure to high levels of thermal stress (Bernard and Kenney, 1994). This stress is not necessarily commensurate with high ambient temperature and or relative humidity (Cheung et al., 2000). The measurement of Tcore is typically achieved using either ear, esophageal, gastro-intestinal, or rectal thermometry. However, at least for research purposes, using telemetric ingestible pills to measure gastro-intestinal temperature *in vivo* in the field is recommended given their accuracy and non-invasive nature (Bongers et al., 2015).

The original Ironman triathlon is now known as the Ironman World Championship and is held in Kailua-Kona, Hawaii, USA annually. Thousands of triathletes from across the globe compete to qualify in this coveted race. One of the main characteristics of this event is its high thermal stress conditions including heat, relative humidity, and solar radiation. Stearns et al., 2018 showed how age group triathletes finished the event with an average Tcore (gastro-intestinal) of 38.3°C. However, it remains unclear if well-trained triathletes experience hyperthermia under thermal stress conditions during the marathon portion of the ironman or only at the end of the event. Well-trained athletes are able to achieve higher velocities and therefore generate increased heat production relative to their competitors, but theoretically, these elite athletes possess efficient mechanisms to dissipate heat and maintain Tcore stability.

Regarding performance, a decrease in running speed following attainment of a critically high Tcore has been demonstrated during the marathon portion of the Ironman in recreational triathletes (Laursen et al., 2009). Despite prior investigations, the true relationship between thermal stress conditions, core hyperthermia, and running performance is unknown. The main purpose of the present study was to evaluate Tcore in top-level, well-trained triathletes before, during, and after the marathon portion of Kona Ironman World Championship (Hawaii) under thermal stress conditions and investigate correlations with running performance. The authors hypothesize that despite the rigorous training status and high performance level of participants, triathletes will obtain elevated core temperatures during the marathon and the temperature elevation will impair their running performance.

## Materials and Methods

### Participants

Fifteen trained and experienced male triathletes were recruited by email to participate in this study (Table 1). Volunteers with a previous history of muscle disorder, cardiac, or kidney disease or those taking medications during the prior 2 week period were excluded from the study. Participants were informed of any potential risks associated with the experiment before providing written consent to participate. The research was carried out during the Ironman World Championship 2014 in Kona, HI (USA). Our selection of highly trained and motivated triathletes for this study was based on our experience that competitive triathletes are generally willing and able to withstand considerable discomfort and to exercise until the development of physiological signs of exhaustion. We scored the number of years that the triathletes had participated in a high-volume training program (>750 h/year). Triathletes arrived on Kona Island 10 days before the race to train in race conditions for the purpose of heat acclimatization. Triathletes and their coaches were informed about the experimental procedure, the possible risks, and benefits of the study with the approval of the local committee of ethics and gave written consent to participate, in accordance with the latest rendering of the Declaration of Helsinki, 2008 (Fortaleza, 2013).

**Table 1:** Physical characteristics of participants and ironman official data.

Variable (units)	
Age (year)	36.11 ± 7.36
Height (cm)	1.79 ± 0.04
Body mass (kg)	71.14 ± 7.12
∑ 6 skin folds (mm)	53.83 ± 8.59
Fat mass (%)	8.48 ± 0.85
Ironman experience (yr)	3.78 ± 1.99
Finish time (h:min:s)	10:06:56 ± 0:48:30
Swimming time (h:min:s)	1:05:23 ± 0:04:49
Transition 1 time (min:s)	3:15 ± 0:48
Cycling time (h:min:s)	5:19:26 ± 0:16:38
Transition 2 time (min:s)	4:29 ± 1:05
Marathon time (h:min:s)	3:34:21 ± 0:38:29
Overall rank (position)	471 ± 397
Age group rank (position)	62 ± 56
Time behind overall elite winner (%)	22.8 ± 9.7
Time behind overall age group winner (%)	10.4 ± 9.1

### Experimental Protocol

The triathlon was held on October 11, 2014 in Kona which is situated 2.13 m above the sea level. Mean dry temperature during the event ranged from 24.4 to 30.0°C. Water temperature was approximately 26°C. Average ambient humidity was approximately 60.4%. Due to the segments of the course where triathletes were most exposed to sun radiation, such as the cycle and run portions, wet bulb globe temperature (WBGT) measurements were calculated (Liljegren et al., 2008; Lemke and Kjellstrom, 2012) according to meteorological data retrieved from the National Weather Service (National Oceanic and Atmospheric Administration, USA).

Mean WBGT during the entire Ironman competition was 24.1°C (range 18.6–30.2°C), while during the marathon portion mean WBGT was 24.7°C (range 22.4–28.5°C). Given these parameters, race officials advised participants to hydrate ad libitum and exercise at their own pace.

#### Before the Race

The race start time was 6:45 am. Three hours before the start of the race (3:45 am local time), participants arrived at a zone near the starting line (main lobby at the official race hotel, the King Kamehameha’s Kona Beach Hotel). Investigators had not provided participants with prior instructions about pre-exercise hydration or nutrition. Nevertheless, all study participants indicated they had consumed breakfast at least 1 hour prior to arriving at the start line. Upon arrival, each participant received an ingestible telemetry pill for the measurement of intra-intestinal temperature (HT150002, HQ^®^ Inc., US). Participants immediately swallowed the pill with 50 ml of water, then rested for 5 min, and completed a survey with two main scales: rating of perceived exertion scale and pain scale. Finally, anthropometric data were obtained. Triathletes were weighed in under garments and all anthropometric measurements were obtained by the same investigator. Investigators did not provide any specific instructions regarding pace, rehydration, or fuel ingestion in order to avoid any undue influence on their routine habits during the race.

#### During the Race

The race consisted of a 3.8 km of open water swim in Kailua-Kona Bay, a 180 km cycle ride across the Hawaiian lava desert (elevation: 1,090 m) and a 42.2 km run along the coast and roads close to the airport. During the race the telemetric sensor registered Tcore.

#### After the Race

Within 1 h of the end of the race, participants proceeded to a secure finish area. Participants were instructed to avoid ingesting fluids from the time they crossed the finish line until the post-race weigh-in where an investigator could assure compliance. Body mass was immediately measured using the same apparatus and methodology employed prior to the race. During this time, scales for ratings of perceived exertion and pain were also completed. The entire post-race process was completed in less than 3 min. Afterward, participants were provided with fluid (water and sports drink) *ad libitum*. At 1 h after finished the race, triathletes left the finish area and moved to corner lobby, official King Kamehameha’s Kona Beach Hotel, where Tcore was registered.

#### Anthropometric Data

Height (cm) was measured by a stadiometer, model SECA^®^ (Germany), with a 2 mm precision and 130–210 cm range. Body mass (kg) was recorded by a scale, model SECA^®^ (Germany), with a precision of 0.2 kg and a range from 2 kg to 130 kg. Body fat percentage was estimated by measuring skinfold thickness (locations: subscapular, tricipital, suprailiac, abdominal, thigh and lower leg) using skinfold calipers (Harpenden, British Indicators, LTD), with an accuracy of 0.2 mm following “The International Society for the Advancement of Kinanthropometry” (ISAK) protocol (Stewart et al., 2011). Additionally, all anthropometric measurements were recorded by the same investigator, who was certified in anthropometric testing (ISAK level 2) (Stewart et al., 2011). Body mass index (BMI) was calculated, and the body mass change attained during the race was calculated as a percent reduction in BMI (pre-to post-race) given that height was constant.

#### Perception Scales

Borg rating of perceived exertion scale (RPE scale) was used to assess participants RPE, where six was equivalent to no exertion and 20 denoted the maximum (Borg, 1982). Visual Analog Scale (VAS pain) was employed to determine the muscular pain as perceived by the subjects in a 90° knee-bending position after trials. Zero on the scale represented no pain experienced, while 10 signified that the movement was extremely painful. This evaluation method has been used in other studies as a non-invasive means of measuring the degree of subjective agreement with certain attitudes or characteristics, such as pain or discomfort (Hicks et al., 2001).

#### Core Temperature

Core body temperature (Tcore) was measured by a telemetric temperature sensor (CorTempTM Ingestible Thermometer^®^, HQInc. USA) using an ingestible pill telemetry system (Byrne and Lim, 2007). Tcore signals were collected and recorded (CorTempTM 2000 Recorder^®^; HQInc. USA) 15 min before the start of the competition (PRE), during the marathon close to km 16 (PER), and 1 h after finishing the ironman (POST).

The manufacture’s reported sensitivity ranges from 0 to 50°C with an accuracy of ±0.1°C. A low-frequency radio wave is transmitted to an external receiver/data logger from a crystal quartz oscillator contained within each pill. The ingestible pill was swallowed 3 h before the race to ensure passage past the stomach, rendering it insensitive to swallowed hot or cold liquids (sensor pills ingested immediately prior to physical activity cannot be used to measure core body temperature accurately in all individuals over the following 13 hours if cool fluids are regularly ingested) (Wilkinson et al., 2008). The calibration of the ingestible pills was verified at four different temperatures against a certified mercury thermometer in a water bath at temperatures ranging from 30 to 42°C. A linear regression of the relationship between the measured temperatures and those from the certified thermometer was used after the test to adjust pill measurements (Edwards and Clark, 2006).

#### Performance and Official Time

The official finish times and the partial times of each of the race components were obtained from the official page of the Kona Ironman World Championship (Hawaii)^1^.

### Statistical Analyses

Data are presented as the mean ± standard deviation (SD). Standard statistical methods were used for the calculation of the mean and SDs. Shapiro-Wilk test (*n* < 50) was conducted to show the distribution of the studied variables and Levene’s test was used for homogeneity of variance. A *t*-test was used to analyze the paired data. A one-way ANOVA *post hoc* Bonferroni test was applied for Tcore analysis. A Pearson’s correlation coefficient was used to test the statistical relationship between different variables. The Cohen’s *d* was calculated to determine the effect size (ES) of the differences with thresholds considered as trivial (<0.2), small (0.2–0.6), moderate (0.6–1.2), large (1.2–2.0), or very large (>2.0) (Cohen, 1988). The differences were considered statistically significant when *p* < 0.05 [SPSS statistical package, version 25.0 was used (SPSS, Inc., Chicago, IL, USA)].

## Results

Table 2 shows dependent variables used in the study in comparison PRE vs. POST. The BMI of participants decreased an average of 4.9% after the ironman reaching statistical significance and with a notable size effect. Perceptual variables, perceived exertion, and perceived muscle pain all increased after finishing the ironman, approaching the highest values of their respective scales. These changes were statistically significant with a moderate and large size effect, respectively.

**Table 2:** BMI and perceptual variables changes pre/post competition.

Variable (units)	PRE	POST	Δ (%)	*p*	ES *d* Cohen
BMI (kg/m^2^)	22.85 ± 1.11	21.73 ± 1.36	−4.9	0.056	3.2 *Very Large*
RPE (units)	10.2 ± 1.64	18.60 ± 1.67	+82.4	0.003	1.1 *Moderate*
VAS pain 0–10 (units)	2.75 ± 1.59	9.08 ± 1.13	+230.2	0.001	2.9 *Very large*

Table 3 describes the results for Tcore of the triathletes. All participants had a normal Tcore before the start of the competition; however, they reached a hyperthermic state during the marathon which persisted at least 1 h after completion of the race. Both the degree of initial increase as well as the degree of persistently elevated temperature in Tcore were statistically significant.

**Table 3:** Core temperature measurement during the ironman.

Variable (units)	PRE (1)	PER (2)	POST (3)	Δ (1–2) (%)	*p*	ES *d* Cohen	Δ (1–3) (%)	*p*	ES *d* Cohen	Δ (2–3) (%)	*p*	ES *d* Cohen
Core temperature (°C)	36.62 ± 0.17	38.55 ± 0.64	38.65 ± 0.41	+4.99	0.01	1.1 large	+5.54	0.002	1.7 *very large*	+0.52	0.182	0.19 *small*

### Relationships Among Variables

#### Race Time and Age Group Rank

Positive correlations were observed between the final placement of age group and marathon time (*r* = 0.913; *p* = 0.01), final time (*r* = 0.816; *p* = 0.007), body mass (*r* = 0.803; *p* = 0.009,) and post-BMI (*r* = 0.931; *p* = 0.007). Similarly, positive correlations were observed between the final race time and body mass (*r* = 0.742; *p* = 0.022), post-BMI (*r* = 0.911; *p* = 0.012) and with time in marathon portion (*r* = 0.892; *p* = 0.001).

#### Core Temperature

Regarding Tcore during the race, the Pearson’s correlation coefficient demonstrated a relationship between Tcore during marathon and Tcore post-competition (*r* = 0.970; *p* = 0.003). Likewise, Tcore during marathon correlated negatively with position in age group (*r* − 0.949, *p* = 0.051) but not with race time (*r* = −0.817; *p* = 0.183). Additionally, an inverse low relationship was found with skin fold summation (*r* = −0.219; *p* = 0.025).

On the other hand, a negative relationship was seen between Tcore post-competition and position in age group (*r* = −0.936, *p* = 0.064) but not with race time (*r* = −0.809; *p* = 0.191). T core post competition showed no correlation with skin fold summation (*r* = −0.164, *p* = 0.806).

#### Perception Scales

A significant correlation was found between the average race time for the run portion and pre-race pain levels (*r* = 0.788; *p* < 0.01). Similarly, a significant association was described between body mass and pain pre-race (*r* = 0.707; *p* < 0.01).

## Discussion

This study was designed to measure Tcore in high-level and well-trained triathletes, under thermal stress conditions, during the marathon portion of one of the most difficult championships in the world, the Ironman World Championship (Hawaii, Kona 2014), (Laird and Johnson, 2012).

The combined 3.8-km swim, 180-km cycle, and 42.192-km run is often considered one of the world’s most challenging endurance races (Lepers, 2008). Given the ACSM position stand about exertional heat illness during training and competition (Armstrong et al., 2007), the average WBGT during the event falls within a higher risk zone for all competitors, but especially for those who are incompletely acclimatized or possess inadequate fitness. Participants in this study demonstrated overall high levels of fitness, given their qualification for an elite Ironman competition, their low fat mass percentage (Kandel et al., 2014) and because their final race time was almost 1 h faster than other Kona participants in similar studies (Stearns et al., 2018).

Exercising in the heat can produce an excessive increase in body Tcore and the resultant hyperthermia can be detrimental to health and endurance performance (Tan and Lee, 2015). Several studies have previously examined Tcore during ironman events in hot conditions. Laursen et al. found that in the Western Australia Ironman in 2006, triathletes averaged a Tcore of 38.1 ± 0.3°C during the event in warm conditions (23.3°C and 60% relative humidity), averaging a lower Tcore during the marathon portion. During the Kona Ironman, recent studies (Stearns et al., 2018) measured Tcore in triathletes immediately after crossing the finish line. With hotter conditions than in Western Australia, the fastest triathletes achieved core temperatures of 38.3 ± 0.6°C while the slowest athletes remained in normothermia with a Tcore about 37.3°C. However, the investigators did not measure or report Tcore during any part of the actual competition.

Our study, performed in the same location but in a different year, demonstrated how 1 hour after finishing the event, triathletes were in an hyperthermic state with a Tcore of 38.65 ± 0.41°C. This means they likely achieved their highest Tcore just after finishing. The Tcore data in our investigation were higher than in other studies referenced, with the main differences being the hotter conditions (Laursen et al., 2006) and faster finish race time in our study (Stearns et al., 2018). The increased heat generated by elite, top-speed athletes as well as the hot, humid ambient conditions are two factors that like increase Tcore.

Regarding Tcore during the event, in the first part of the marathon, triathletes had reached hyperthermia (38.55 ± 0.64°C) in our study, data that explain the high Tcore reached 1 h after finished the event supported by a high and statistically significant correlation (*r* = 0.970; *p* = 0.003). To our knowledge this work represents the first actual reporting of Tcore measurements obtained during the race of the Ironman World Championships.

In our study, the persistent elevation of Tcore for at least 60 min after the event (38.65 ± 0.41°C, *p* < 0.002) represents a somewhat surprising and novel finding. After cessation of exercise, the rate at which body produces heat decreases while the mechanisms used to dissipate heat remain in operation until the T core returns to its normal level. The effectiveness of the thermoregulatory system in regulating body temperature is influenced by the acclimatization state of the individual (Wenger, 1988). In hot and humid race venues such as the Kona Ironman, many professional triathletes effectively utilize strategies to “beat the heat and humidity” such as training in the heat in the days leading up to the event (Lockett, 2012). For this reason, many triathletes arrive in Kona early and spend 7–10 days training in the heat with the goal of acclimatization leading to improved performance.

The assumption that high internal T core can cause fatigue has been accepted (González-Alonso, 2007) and is supported by a case report in an Ironman triathlon (Laursen et al., 2009). Correlation established in our study shows how a higher Tcore during the marathon is inversely related to age group rank (*r* = −0.949, *p* = 0.051).

During these long endurance events, body mass changes are substantial. Mueller showed that participation in an Ironman event can lead to important changes in body composition such as a substantial loss of fat mass, which can affect energy availability since fat provides up to 50% of whole energy expenditure (Mueller et al., 2013). Knechtle and colleagues showed that male triathletes in an Ironman lose an average of 1.8 kg of body mass and 1 kg of skeletal muscle mass, presumably due to a depletion of stored glycogen and lipids within myocytes (Knechtle et al., 2010). In our study the post-event BMI decreased 4.9% with a very large size effect. Considering that height is stable, the reduction implicates a decrease in body mass, which is similar to findings discovered during the Ironman of South Africa by Hew-Butler et al. (2007). This change in body mass is likely attributed primarily to a loss of fluid. Stearns et al., 2018 found in the Kona Ironman only a 2.4% decrease in body mass, but the participants’ urine specific gravity was 1.021 after finishing the race, implying a significant ingestion of fluids. Unfortunately in our study, we were not able to asses biological markers related to hydration/dehydration status.

Body composition is also related to performance in endurance sports, including triathlons. An excess of body mass may be especially disadvantageous in the run segment (Sleivert and Rowlands, 1996). Some research claims that a loss in body mass during marathon and triathlon events contributes to an athlete’s success, especially for those who lose substantially more than 3–4% body weight during competition.

In our study, positive correlations were found between the final age group rank and final BMI (*r* = 0.931; *p* = 0.007) what might indicate that triathletes who complete the race with a lower BMI, i.e., lower body mass, finish with a higher rank position. To get an optimal position in age group rank, ideally a competitor secures a favorable marathon split (*r* = 0.913; *p* = 0.01). Also to achieve a lower finish time, one must have a lower marathon split time (*r* = 0.892; *p* = 0.001) and a low pre-race body mass (*r* = 0.742; *p* = 0.022). Fast time in running, as well as other factors, has been previously reported as an important predictive variable for a fast race in long distance triathlons (Knechtle et al., 2015).

The results of our study reiterate the importance of a fast marathon pace for achieving a higher rank in one’s age group, due to their positive and strong correlation. A loss of body mass up to 5% may allow an athlete to increase velocity but may not positively affect thermoregulation as a negative relationship was seen between Tcore during marathon and one’s position in age group. Subsequently, cooling strategies during marathon or heat acclimation before the event may enhance performance in triathletes.

In regard to perceptual variables, in sports and particularly exercise testing, the rating of perceived exertion (RPE), as measured by the Borg rating of perceived exertion scale (RPE scale) (Borg, 1982) is frequently used to quantify measures of perceived exertion. An interesting study conducted in Ironman triathletes (Parry et al., 2011) concluded that RPE followed a linear progression during each triathlon discipline followed by a return of the perception of effort to baseline levels at the start of the next discipline. Our results revealed that RPE values increased significantly after the event, which is in concordance with Parry and colleagues, who previously demonstrated that the increase in RPE for the entire event followed a linear pattern. Confounding the results, anxiety and mood responses of participants seem to indicate that the emotional response of athletes before and after ultra-endurance events is closely aligned with their conscious thoughts (Parry et al., 2011). Furthermore, RPE values of triathletes after completing the Ironman World Championship in Kona from this study were very similar to those achieved years before in the same race by a different cohort (Stearns et al., 2018).

Visual Analog Scales (VAS) are psychometric tools. The VAS has been used in other studies as a non-invasive method of monitoring the changes in muscular pain perception after exercising, and the consequent muscle damage. In triathletes from this study, subjective levels of muscle pain increased significantly by 230% after the event with a score of 9.08 ± 1.13. Muscle pain may be severe and may interfere with gait, causing participants to seek assistance in the medical tent after the event. Such discomfort derives from muscle damage which may multifactorial in nature (Gleeson and Bishop, 2000).

Prior studies have assessed pain and plasma muscle enzyme levels as potential markers for muscle damage; however, general consensus indicates that these parameters do not accurately measure the degree of injury to skeletal muscle (Kim et al., 2007). Muscle damage associated with prolonged activities, such as the Ironman triathlon, is associated with increases in creatine kinase (CK) levels (Machado et al., 2010), release of interleukins, and consequently, modulation of the immune system. It has been suggested that immunological and hormone alterations occur due to high-volume and high-intensity training which, combined with the emotional and physiologic stress caused by the competition, may be perceived after the event (Walsh et al., 2011). This fact supports the need for a more detailed examination of athletes <35 years and to identify people who are at risk for muscle damage (Leischik and Spelsberg, 2014).

In summary, hyperthermia is reached during the marathon portion of Ironman World Championship in Kona under thermal stress conditions. Increased core temperature appears to make triathletes run more slowly while a fast marathon split is very important for a good rank in age groups. Also hyperthermia persists 1 h after the event. Cooling strategies during the race and heat acclimation must be utilized for preparing these types of competitions.

One limitation of this study was that Tcore was registered only in three separate moments. A full register of Tcore during the entire event may provide additional information about Tcore alterations and timing of reaching hyperthermia, whether during the marathon or before. Another limitation was the inability to perform blood/urine analysis to obtain biological markers for a more complete, multifactorial analysis of results obtained.

Future research regarding to this world championship should focus on continuous monitoring of Tcore in order to identify the degree of hyperthermia experienced by triathletes or the effects of different cooling strategies during the race.

## Data Availability Statement

All datasets generated for this study are included in the article/supplementary material.

## Ethics Statement

The studies involving human participants were reviewed and approved by the University of Extremadura. The patients/participants provided their written informed consent to participate in this study.

## Author Contributions

GO conceptualized the study. GO, CC, JC-G, and RT assisted with the methodology. GO, CC, and JC-G acquired measurements and assisted with the data field acquisition. GO, RT, and JC-G contributed in formal analysis. GO, JM, and JC-G assisted with the writing and with the original draft preparation. GO, CC, RT, JM, and JC-G assisted with the writing, review, and editing.

### Conflict of Interest

The authors declare that the research was conducted in the absence of any commercial or financial relationships that could be construed as a potential conflict of interest.

## Footnotes

^1^www.eu.ironman.com/triathlon/event
